# The impact of finasteride and dutasteride treatments on proliferation, apoptosis, androgen receptor, 5α-reductase 1 and 5α-reductase 2 in TRAMP mouse prostates

**DOI:** 10.1016/j.heliyon.2017.e00360

**Published:** 2017-07-24

**Authors:** Alexander B. Opoku-Acheampong, Jamie N. Henningson, Brian L. Lindshield

**Affiliations:** a Department of Food, Nutrition, Dietetics and Health, Kansas State University, Manhattan, KS 66506, USA; b College of Veterinary Medicine, Kansas State University, Manhattan, KS 66506, USA

**Keywords:** Pathology, Cell biology, Physiology, Oncology, Cancer research, Anatomy

## Abstract

**Background:**

Previously, we studied the effect of finasteride- or dutasteride-containing diets in male C57BL/6 TRAMP x FVB mice. Pre (6 weeks of age) and post (12 weeks of age) groups received finasteride or dutasteride to determine the efficacy of these pharmaceuticals on prostate cancer (PCa) development in male C57BL/6 TRAMP x FVB mice. Post-Dutasteride treatment was more effective than Pre-Dutasteride treatment, and dutasteride treatments were more effective than finasteride treatments in decreasing prostatic intraepithelial neoplasia (PIN) progression and PCa development. Finasteride and Pre-Dutasteride treatments significantly decreased high-grade PIN incidence, but increased poorly differentiated PCa incidence. In this study, molecular changes in prostates of these mice were characterized in an effort to elucidate the discordant response in Pre-Dutasteride and finasteride groups, and determine why Post-Dutasteride treatment was more effective.

**Method/Principal findings:**

Ki-67 (proliferation marker) and androgen receptor (AR) protein, apoptotic DNA fragmentation (TUNEL assay), 5α-reductase 1 (5αR1) and 5α-reductase 2 (5αR2) mRNA were quantified in male TRAMP mice prostate tissues with genitourinary weight < 1 and > 1 gram. Overall, proliferation and AR were decreased and apoptosis was increased in most tumors versus prostate epithelium and hyperplasia. Proliferation and AR were increased notably in hyperplasia versus prostate epithelium and tumor. There were no clear trends or differences in 5α-reductase 1 and 5α-reductase 2 levels between large and small tumors. The discordant response in Pre-Finasteride and Pre-Dutasteride groups may be due to upregulated 5αR1 levels in large versus small tumors. It is not clear what the mechanism is for the different response in the Post-Finasteride group. Post-Dutasteride treatment was more effective than Pre-Dutasteride treatment in decreasing 5αR1 in large tumors. Therefore, this may be why this treatment was more effective in decreasing PIN progression and PCa development.

**Conclusion:**

The effect of finasteride and dutasteride on these biomarkers did not clearly elucidate their mechanism of action, but tumor 5αR1 levels were significantly positively correlated with adjusted prostate severe lesion score.

## 1 Introduction

Prostate cancer is the most commonly diagnosed male cancer in the US and the third leading cause of male cancer death [1]. Most prostate cancer growth is androgen-dependent or sensitive at the initial stages of development [2]. In prostate stromal cells, testosterone, the main plasma androgen is converted by 5α-reductase 1, 5α-reductase 2 and 5α-reductase 3 isoenzymes to the more potent dihydrotestosterone (DHT), which binds with up to 10-fold higher affinity to the androgen receptor (AR) than testosterone [3, 4, 5, 6]. 5α-reductase 2 is predominantly expressed in prostate tissues [7], however, several [8, 9, 10, 11], although not all [12, 13], studies have reported increased/unchanged 5α-reductase 1 and/or decreased/lost 5α-reductase 2 mRNA expression/activity in prostate cancer versus nonmalignant prostate tissues. Recently, 5α-reductase 3 has been identified as a new isoenzyme and found to be overexpressed in hormone-refractory prostate cancer cells and tissues [5].

In the adult prostate, androgens contribute to the maintenance of homeostasis between cell proliferation and apoptosis [14, 15]. In prostate luminal epithelium, DHT binds to ARs to trigger the transcription of cell proliferation and apoptosis control genes by regulating the expression and secretion of growth factors. Animal studies have shown that prostatic stromal cells secrete growth factors that act on epithelial cells to modulate proliferation and apoptosis [14, 16, 17]. Higher prostate epithelium AR levels increase the progression to castration-resistant prostate cancer in men [18], proliferative rate, and prostate cancer risk in rat probasin-murine AR (Pb-mAR) transgenic mice [19]. AR is expressed in almost all primary prostate cancers and most castration-resistant prostate cancers [20, 21], however, AR expression loss in prostate tumor cells has been reported [22]. In humans, DHT may induce the production of epidermal growth factor (EGF), keratinocyte growth factor (KGF), and insulin-like growth factors (IGFs)—all of which stimulate cellular proliferation [14]. Similarly, DHT subdues the capacity of transforming growth factor-β (TGF-β) to induce apoptosis of prostatic epithelial cells [17, 23]. In prostate cancer, cell proliferation and apoptosis dysregulation leads to an imbalance between cell division and cell death, which collectively contribute to tumorigenesis and tumor progression [24].

Cell proliferation and apoptosis are androgen-dependent mechanisms in prostate cancer [14, 15], therefore decreasing DHT production via 5α-reductase inhibition may be effective in preventing, or delaying, the growth of prostate cancer [25]. Finasteride (5α-reductase 2 inhibitor) and dutasteride (5α-reductase 1 and 5α-reductase 2 inhibitor) are two pharmaceuticals commonly used to treat benign prostatic hyperplasia. The potential of these inhibitors to decrease prostate cancer development and/or progression through their antiandrogen action has been investigated in several clinical trials [26, 27, 28].

Previously, initiation of finasteride- or dutasteride-containing diets (83.3 mg/kg of diet) at 6 weeks (Pre) or 12 weeks (Post) of age in male C57BL/6 TRAMP x FVB mice resulted in Post-Dutasteride treatment being more effective than Pre-Dutasteride treatment, and both dutasteride treatments being more effective than both finasteride treatments in decreasing prostatic intraepithelial neoplasia (PIN) progression and prostate cancer development. Finasteride and Pre-Dutasteride treatments also increased the incidence of poorly differentiated prostate cancer in most lobes versus control [29]. Thus, characterizing the molecular changes in the prostates of these mice may elucidate the discordant response in the Pre-Dutasteride and finasteride groups and determine why Post-Dutasteride treatment was more effective.

To this end, cell-type specific expression patterns of Ki-67 and AR protein, apoptotic DNA fragmentation, 5α-reductase 1 and 5α-reductase 2 mRNA levels were determined in formalin-fixed, paraffin-embedded prostate tissue sections of finasteride and dutasteride treated male C57BL/6 TRAMP x FVB mice. These prostate cancer biomarkers were selected because of their relationship to the expected response to 5α-reductase inhibition. We compared prostate cancer biomarker levels in mice with genitourinary (GU; composed of seminal vesicles, prostate and urinary bladder) weight < 1 gram (associated with lower most common and most severe lesion scores) to levels in mice with GU weight > 1 gram (associated with higher most common and most severe lesion scores).

## 2 Materials and methods

### 2.1 Ethics statement

The Institutional Animal Care and Use Committee (IACUC) at Kansas State University approved all animal procedures (protocol 2969).

### 2.2 Tissues

Prostate tissue sections (4 μm) from male C57BL/6 TRAMP x FVB mice in AIN–93 G control, Pre-Finasteride (Pre-F), Post-Finasteride (Post-F), Pre-Dutasteride (Pre-D) and Post-Dutasteride (Post-D) diet groups from our previous study [29] were used for immunohistochemistry (IHC) and *in situ* hybridization (ISH) analysis in this study. One slide per animal was used to quantify each biomarker. At the time of euthanasia, a majority of prostate samples were completely wiped out by tumor. Hence prostate lobes could not be identified in most samples.

### 2.3 Histopathology, IHC, ISH, and biomarker quantification

**Fig. 1:**
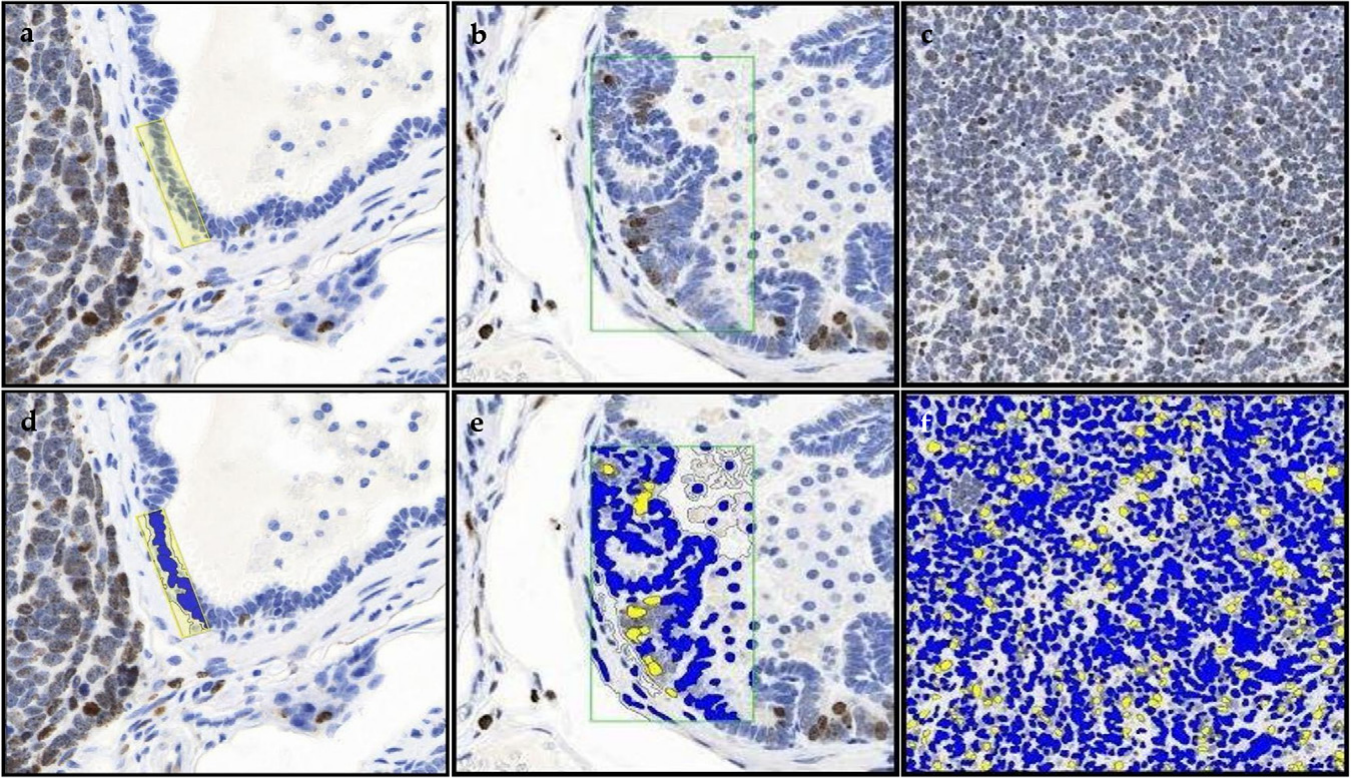
Representative staining for Ki-67 in (a) prostate epithelium showing single layer of cells, (b) hyperplasia showing focal increased cell density with piling up on one another, and (c) tumor showing diffuse sheets of cells with no organization and characterized by neoplastic cellular characteristics, captured at 2X magnification. The rectangular boxes within the different tissue specific cell types are representative sections that were digitized (annotated) with Halo software to identify and quantify Ki-67 immunopositive staining in (d) prostate epithelium, (e) hyperplasia, and (f) tumor.

Histological processing of prostate tissues and sectioning were performed as described previously [29]. The expression levels of Ki-67 and AR protein, apoptotic DNA fragmentation were quantified using IHC; and 5α-reductase 1 and 5α-reductase 2 mRNA were quantified using ISH in formalin-fixed, paraffin-embedded prostate tissue sections of AIN–93 G control, Pre-Finasteride, Post-Finasteride, Pre-Dutasteride, and Post-Dutasteride (n = 5) treated male TRAMP mice with GU weight < 1 gram; and AIN–93 G control, Pre-Finasteride, Post-Finasteride (n = 4), Pre-Dutasteride (n = 2), and Post-Dutasteride (n = 4) treated male TRAMP mice with GU weight > 1 gram. Biomarker assays and quantification in prostate epithelium, hyperplasia and tumor were performed as described previously [30]. Thresholds for positivity were defined so that they matched what was seen visually. This was done by looking at images across the range of intensities so that the established thresholds best fit the entire group rather than shifting the threshold image by image. Prostate epithelium was defined as histologically normal prostate. Biomarker prostate epithelium, hyperplasia and tumor representative staining images in Figs. 1, 3, 5 and 7 were captured at 2X magnification.

### 2.4 Statistical analysis

Data were analyzed using ANOVA with Fisher’s Least Significant Difference (LSD) as described previously [30] using SAS 9.4 (SAS Institute Inc., Cary, NC) with *p* < 0.05 considered statistically significant. Cell types with n < 2 were excluded from statistical analysis, and the modified Thompson tau technique was used to eliminate outliers. Correlation between the biomarkers measured and adjusted prostate lesion score severity was analyzed using the Spearman rank correlation coefficient (r).

## 3 Results

### 3.1 Cell proliferation

**Fig. 2:**
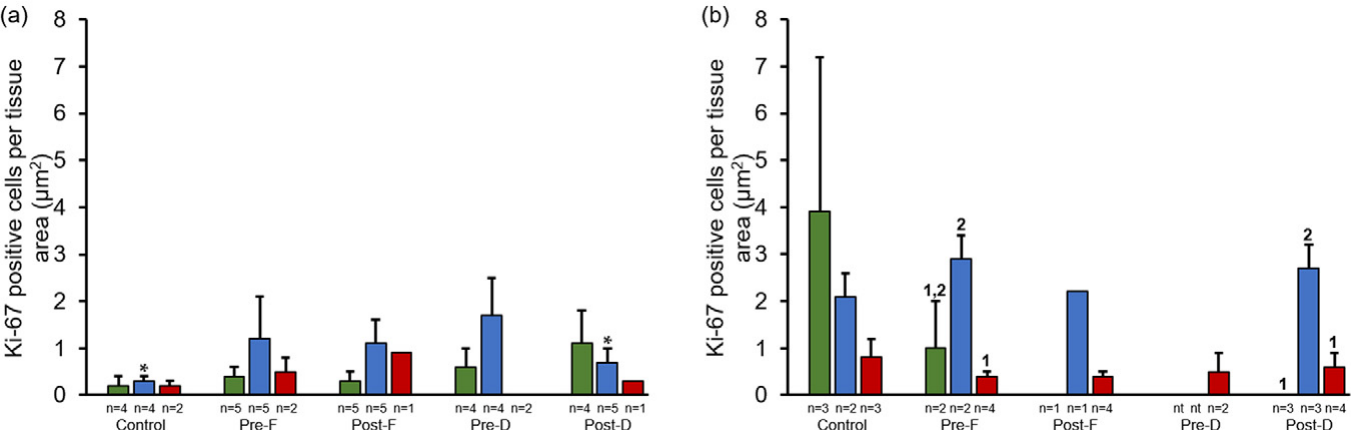
Ki-67 expression in finasteride and dutasteride treated TRAMP mice with GU weight < 1 (a) and > 1 (b) gram. The values are the mean total Ki-67 positive cells per tissue area (μm^2^) ± SEM in prostate epithelium, hyperplasia, or tumor. Data are multiplied by 1000. Bars with different numbers are statistically different from one another within group (*p* < 0.05). Bars with asterisk are statistically different from one another between cell type within group in GU weight < 1 (a) vs. > 1 (b) gram, respectively (*p* < 0.05). nt = no cell type was identified within tissue. Green = Prostate epithelium; Blue = Hyperplasia; Red = Tumor.

Ki-67 prostate epithelium, hyperplasia and tumor representative staining, and their annotated images are shown in Fig. 1a-f. There were no significant differences in Ki-67 expression between treatment groups (Fig. 2a-b; Table 1 in the Appendix). There was a significant decrease in Ki-67 expression in hyperplasia of the control and Post-Dutasteride groups with GU weight < 1 gram versus respective groups with GU weight > 1 gram. In Pre-Finasteride mice with GU weight > 1 gram, there was a significant increase in Ki-67 expression in hyperplasia versus tumor, and a significant increase in Ki-67 expression in hyperplasia versus prostate epithelium and tumor of the Post-Dutasteride group.

### 3.2 Apoptosis

**Fig. 3:**
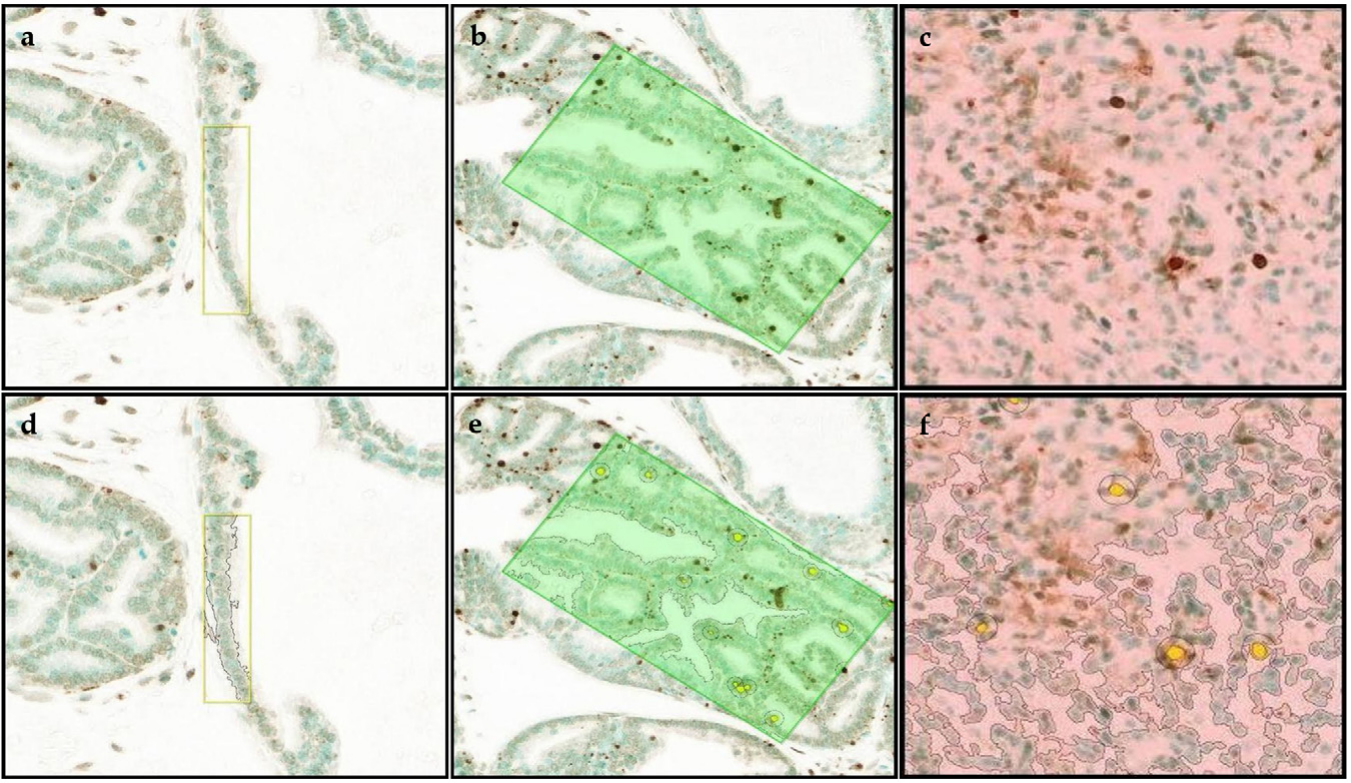
Representative TUNEL staining in (a) prostate epithelium showing single layer of cells, (b) hyperplasia showing focal increased cell density with piling up on one another, and (c) tumor showing diffuse sheets of cells with no organization and characterized by neoplastic cellular characteristics, captured at 2X magnification. The rectangular boxes within the different tissue specific cell types are representative sections that were digitized (annotated) with Halo software to identify and quantify TUNEL immunopositive staining in (d) prostate epithelium, (e) hyperplasia, and (f) tumor.

**Fig. 4:**
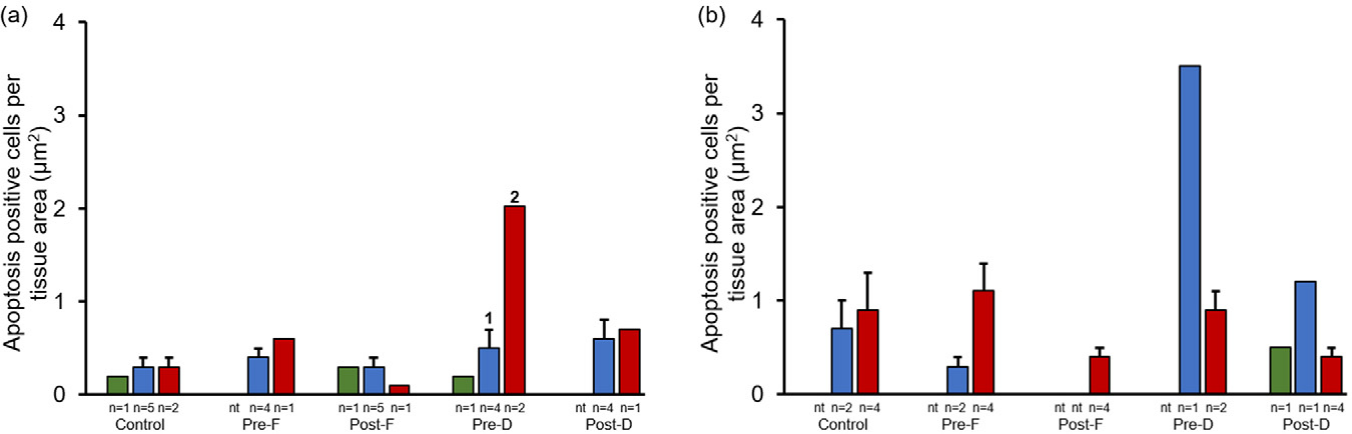
Apoptosis expression in finasteride and dutasteride treated TRAMP mice with GU weight < 1 (a) and > 1 (b) gram. The values are the mean total apoptosis positive cells per tissue area (μm^2^) ± SEM in prostate epithelium, hyperplasia, or tumor. Data are multiplied by 1000. Bars with different numbers are statistically different from one another within group (*p* < 0.05). nt = no cell type was identified within tissue. Green = Prostate epithelium; Blue = Hyperplasia; Red = Tumor.

Apoptosis prostate epithelium, hyperplasia and tumor representative staining, and their annotated images are shown in Fig. 3a-f. There were no significant differences between treatment groups or between GU weight subgroups (Fig. 4a-b; Table 2 in the Appendix). Apoptosis was significantly increased in tumor versus hyperplasia of the Pre-Dutasteride group with GU weight < 1 gram.

### 3.3 Androgen receptor

**Fig. 5:**
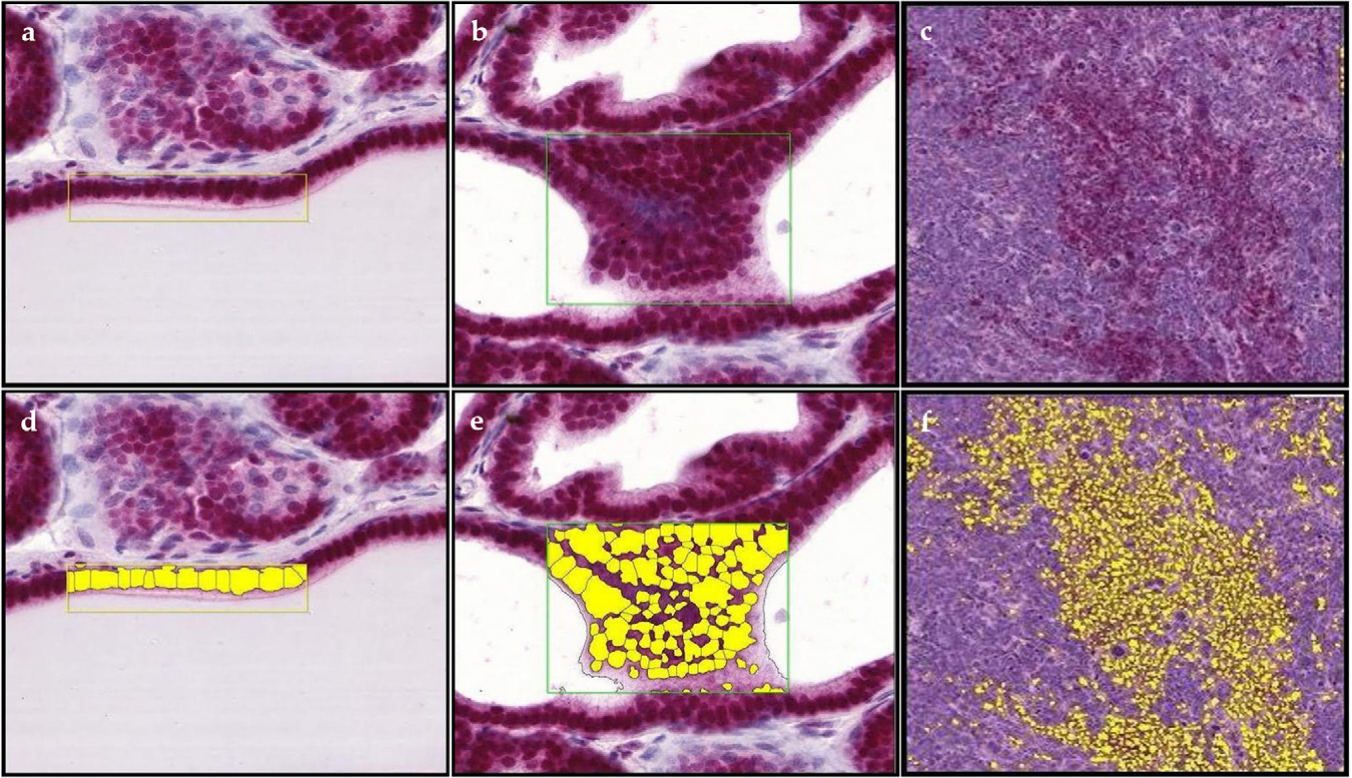
Representative staining for androgen receptor in (a) prostate epithelium showing single layer of cells, (b) hyperplasia showing focal increased cell density with piling up on one another, and (c) tumor showing diffuse sheets of cells with no organization and characterized by neoplastic cellular characteristics, captured at 2X magnification. The rectangular boxes within the different tissue specific cell types are representative sections that were digitized (annotated) with Halo software to identify and quantify androgen receptor immunopositive staining in (d) prostate epithelium, (e) hyperplasia, and (f) tumor.

**Fig. 6:**
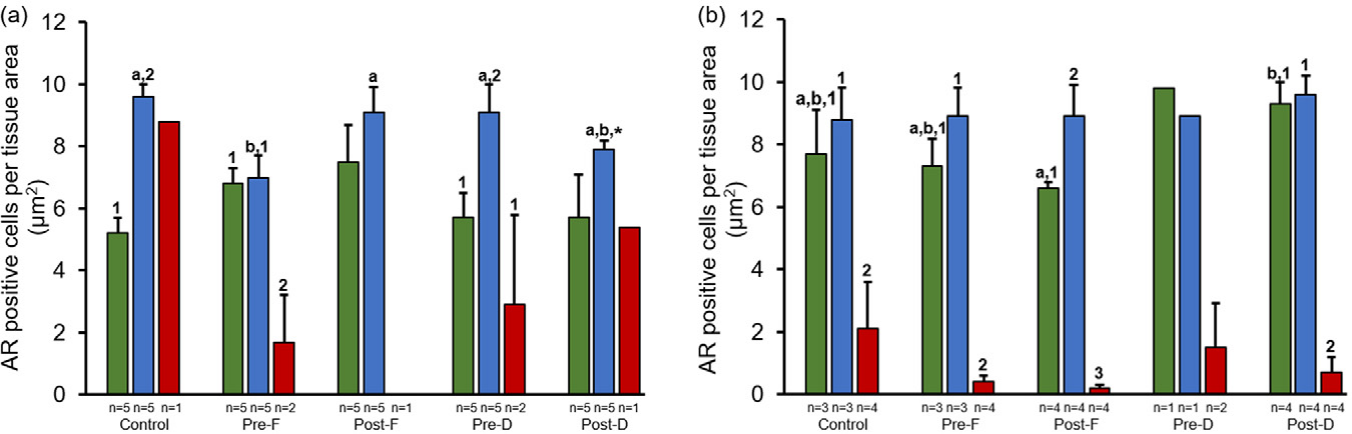
Androgen receptor (AR) expression in finasteride and dutasteride treated TRAMP mice with GU weight < 1 (a) and > 1 (b) gram. The values are the mean total androgen receptor positive cells per tissue area (μm^2^) ± SEM in prostate epithelium, hyperplasia, or tumor. Data are multiplied by 1000. Bars with different letters are statistically different from one another between groups (*p* < 0.05). Bars with different numbers are statistically different from one another within group (*p* < 0.05). Bars with asterisk are statistically different from one another between cell type within group in GU weight < 1 (a) vs. > 1 (b) gram, respectively (*p* < 0.05). Green = Prostate epithelium; Blue = Hyperplasia; Red = Tumor.

AR prostate epithelium, hyperplasia and tumor representative staining, and their annotated images are shown in Fig. 5a-f. There was a significant decrease in AR expression in hyperplasia of the Pre-Finasteride group versus all except the Post-Dutasteride group with GU weight < 1 gram (Fig. 6a; Table 3 in the Appendix). AR expression in hyperplasia was significantly decreased in the Post-Dutasteride group with GU weight < 1 gram versus Post-Dutasteride group with GU weight > 1 gram. In mice with GU weight < 1 gram, AR expression was significantly increased in hyperplasia versus prostate epithelium of the control and in prostate epithelium and hyperplasia versus tumor of the Pre-Finasteride group. AR expression in hyperplasia versus prostate epithelium and tumor was significantly increased in the Pre-Dutasteride group with GU weight < 1 gram. In mice with GU weight > 1 gram, tumor AR expression was significantly decreased versus prostate epithelium and hyperplasia of the control, Pre-Finasteride, Post-Finasteride, and Post-Dutasteride groups (Fig. 6b). AR was significantly increased in hyperplasia versus prostate epithelium of the Post-Finasteride group with GU weight > 1 gram. In mice with GU weight > 1 gram, prostate epithelium AR expression was significantly increased in the Post-Dutasteride versus Post-Finasteride group.

### 3.4 5α-reductase 1 and 5α-reductase 2

**Fig. 7:**
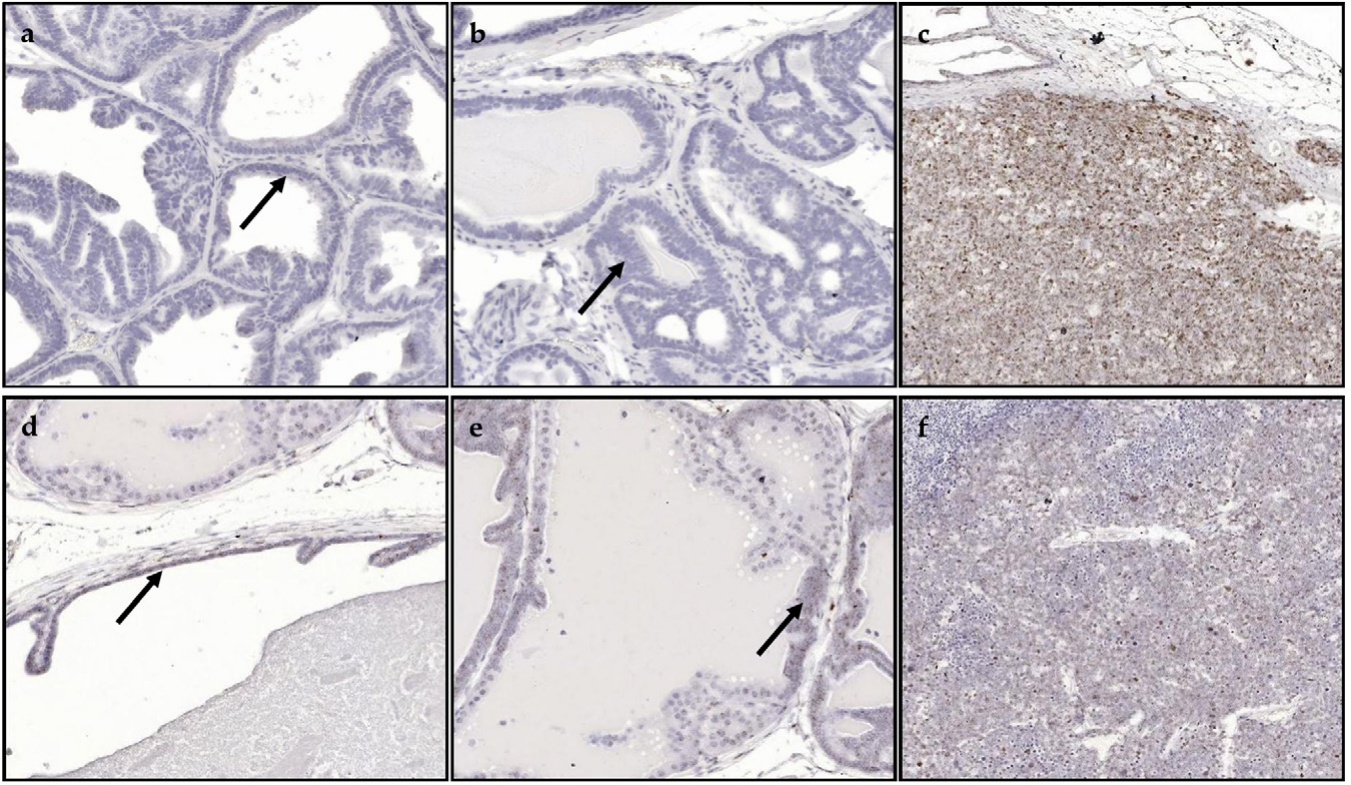
Representative staining for 5α-reductase 1 and 5α-reductase 2 mRNA (a and d, respectively) in prostate epithelium showing single layer of cells, (b and e, respectively) hyperplasia showing focal increased cell density with piling up on one another, and (c and f, respectively) tumor showing diffuse sheets of cells with no organization and characterized by neoplastic cellular characteristics, captured at 2X magnification. The arrows within select tissues are representative sections showing 5α-reductase 1 and 5α-reductase 2 mRNA staining.

**Fig. 8:**
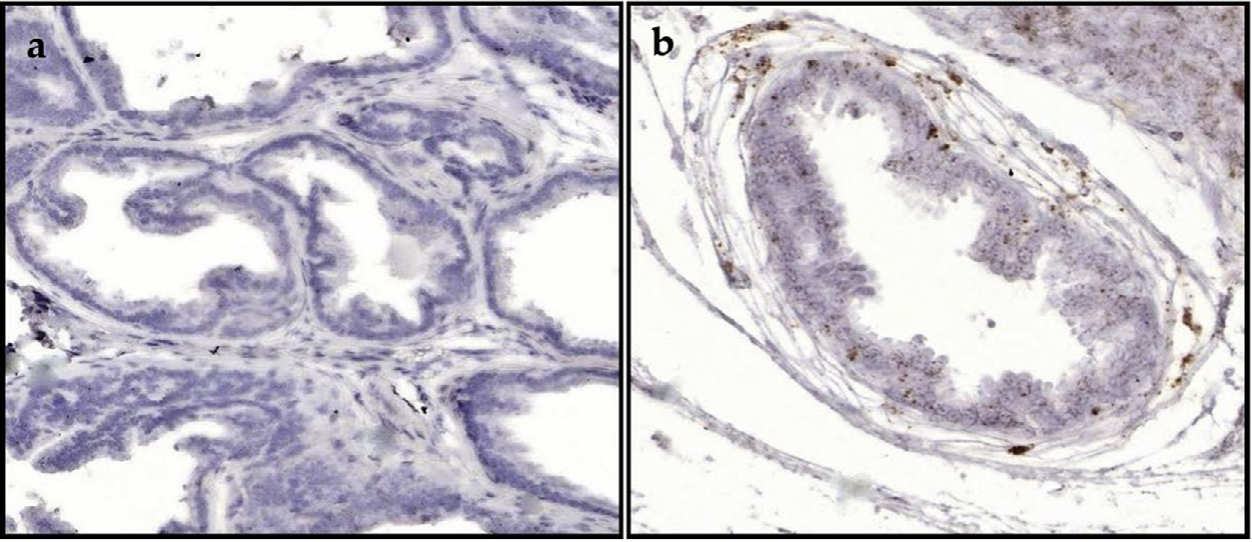
Representative staining for 5α-reductase 1 (a) and 5α-reductase 2 (b) mRNA in prostate stroma, which is composed of smooth muscle cells, fibroblasts, myofibroblasts, endothelial cells and immune cells, captured at 2X magnification.

**Fig. 9:**
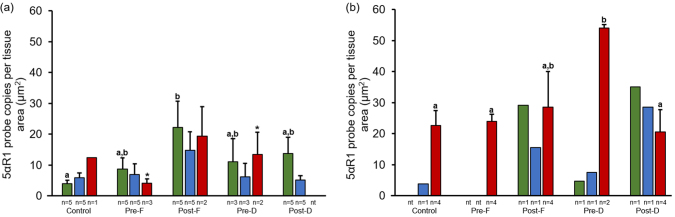
5α-reductase 1 expression in finasteride and dutasteride treated TRAMP mice with GU weight < 1 (a) and > 1 (b) gram. The values are the mean total 5α-reductase 1 probe copies per tissue area (μm^2^) ± SEM in prostate epithelium, hyperplasia, or tumor. Data are multiplied by 1000. Bars with different letters are statistically different from one another between groups (*p* < 0.05). Bars with asterisk are statistically different from one another between cell type within group in GU weight < 1 (a) vs. > 1 (b) gram, respectively (*p* < 0.05). nt = no cell type was identified within tissue. Green = Prostate epithelium; Blue = Hyperplasia; Red = Tumor.

**Fig. 10:**
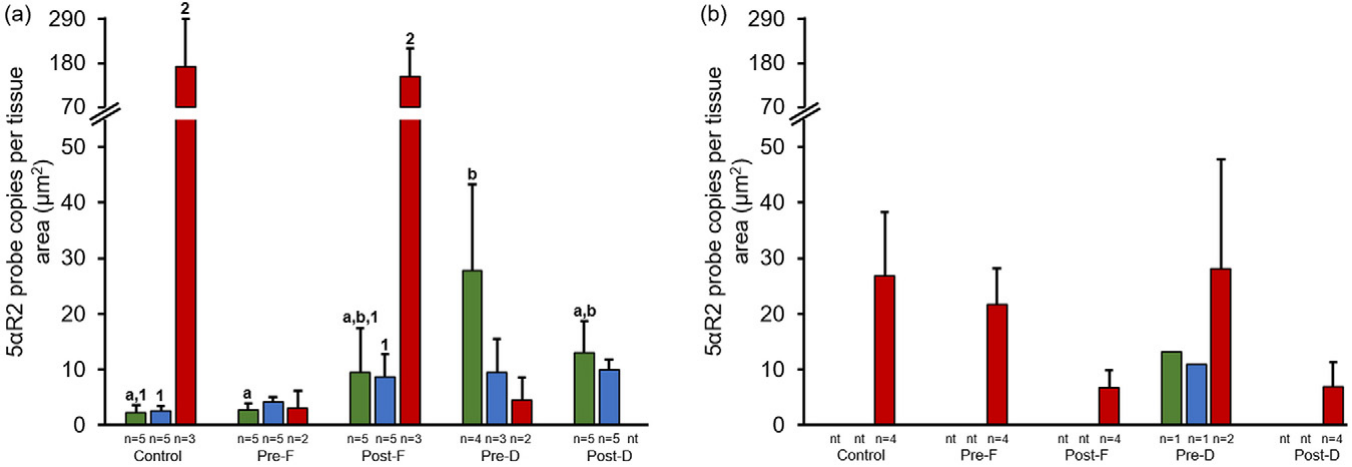
5α-reductase 2 expression in finasteride and dutasteride treated TRAMP mice with GU weight < 1 (a) and > 1 (b) gram. The values are the mean total 5α-reductase 2 probe copies per tissue area (μm^2^) ± SEM in prostate epithelium, hyperplasia, or tumor. Data are multiplied by 1000. Bars with different letters are statistically different from one another between groups (*p* < 0.05). Bars with different numbers are statistically different from one another within group (*p* < 0.05). nt = no cell type was identified within tissue. Green = Prostate epithelium; Blue = Hyperplasia; Red = Tumor.

5α-reductase 1 and 5α-reductase 2 prostate epithelium, hyperplasia and tumor representative staining are shown in Fig. 7a-f. 5α-reductase 1 was more frequently expressed in prostate epithelium versus stroma. 5α-reductase 2 was predominantly expressed in prostate epithelium and stroma. Representative staining for 5α-reductase 1 and 5α-reductase 2 mRNA in prostate stroma are shown in Fig. 8a and b, respectively. In mice with GU weight < 1 gram, 5α-reductase 1 levels were significantly increased in prostate epithelium of the Post-Finasteride group versus the control group (Fig. 9a; Table 4 in the Appendix). In mice with GU weight > 1 gram, 5α-reductase 1 levels were significantly increased in tumor of the Pre-Dutasteride group versus all groups except the Post-Finasteride group (Fig. 9b). There was a significant decrease in 5α-reductase 1 levels in tumor of the Pre-Finasteride and Pre-Dutasteride groups with GU weight < 1 gram versus their respective treatment groups with GU weight > 1 gram. 5α-reductase 2 levels were significantly increased in prostate epithelium of the Pre-Dutasteride group versus the control and Pre-Finasteride groups with GU weight < 1 gram (Fig. 10a; Table 5 in the Appendix). 5α-reductase 2 levels were significantly increased in tumor versus prostate epithelium and hyperplasia of the control and Post-Finasteride groups with GU weight < 1 gram. There were no significant differences in 5α-reductase 2 levels in tumor between treatment groups with GU weight > 1 gram (Fig. 10b).

### 3.5 Biomarker correlation with adjusted prostate lesion score severity

**Fig. 11:**
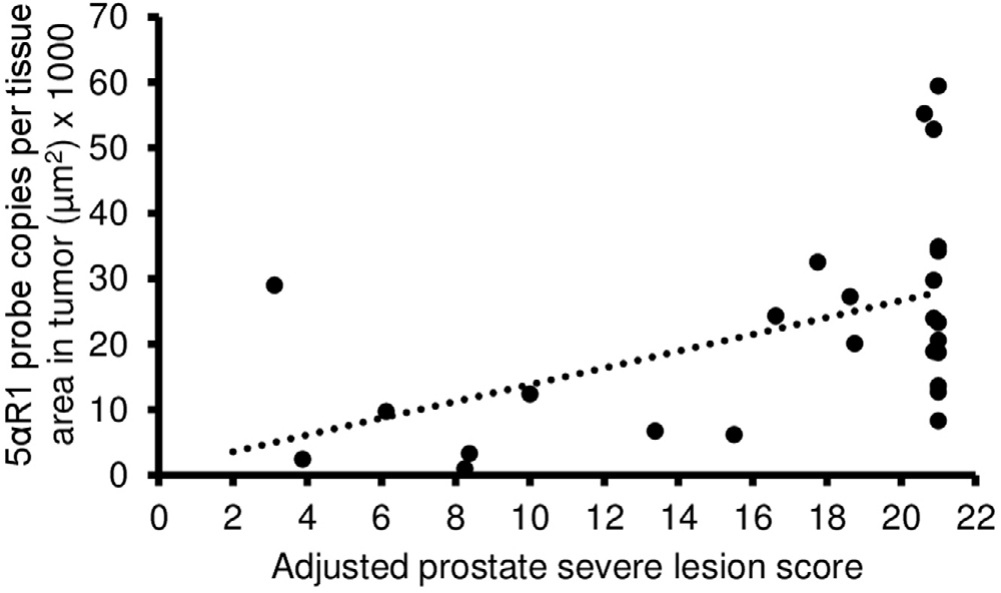
Spearman correlation coefficient (r) between 5α-reductase 1 (5αR1) in tumor and adjusted prostate lesion score severity. There was a significant positive correlation between 5α-reductase 1 in tumor and adjusted prostate lesion score severity (r = 0.41; *p* = 0.04).

Only one significant correlation between biomarkers and adjusted prostate lesion score severity was identified. Adjusted prostate lesion score severity was used because it combines lesion severity with an indication of its distribution within a lobe [31]. Tumor 5α-reductase 1 levels were significantly positively correlated with adjusted prostate lesion score severity (Fig. 11). Similar correlation trends were noted across treatment groups suggesting that treatment did not impact this correlation.

## 4 Discussion

There was no significant difference in prostate epithelium, hyperplasia and tumor cell proliferation between groups, except the significant increase in the Post-Dutasteride GU weight > 1 gram hyperplasia. However, this group’s Ki-67 levels were similar to those seen in other groups’ hyperplasia with tumor > 1 gram. The overall lack of difference in treatment groups may suggest that regulation of proliferation was not a major mechanism through which Post-Dutasteride treatment results in improved outcomes. GU weight < 1 gram tumor proliferation rates are consistent with similar findings reported previously following androgen withdrawal in mice bearing the CWR22 human prostate cancer xenograft [32, 33, 34], dutasteride treatment in men with prostate cancer [35], and castration in both TRAMP mice [36] and human prostatic tumors [37]. 5α-reductase inhibitors cause histological changes in prostate similar to those caused by androgen ablation therapy, and milder than those caused by androgen withdrawal [38].

The presence of neuroendocrine features in TRAMP prostate cancer cells allows them to escape apoptosis [39], enhance their malignant potential via resistance to drug and hormonal therapies [40], which contradicts the overall nonsignificant difference in apoptosis in tumor versus prostate epithelium or hyperplasia seen in this study. This is also not consistent with increased apoptosis in human prostate cancer following androgen withdrawal [41], LPB-Tag mice dorsolateral [42] and human prostate following dutasteride treatment [35], and castrated C57BL/6 TRAMP mice prostate [36]. Previous studies found that finasteride shrunk prostate volume and caused epithelial involution through induction of apoptosis to inhibit tumor growth [43, 44]. Decreased apoptosis in prostate epithelium is not consistent with previous evidence [44] in men following finasteride treatment. It is not clear what the cause is for this different response. Overall, finasteride and dutasteride may decrease apoptosis in prostate epithelium, but stimulate it in hyperplasia and tumor.

Androgens acting through ARs act both as agonistic stimulators of cell division and as antagonistic inhibitors of cell death [15]. Finasteride and dutasteride treatments resulted in nonsignificantly decreased AR expression in tumors in most groups, which is not consistent with increased AR expression in castrated TRAMP mice small and large tumors [45], tumors from men who received androgen deprivation therapy for prostate cancer [46], and LNCaP human prostate cancer cells treated with finasteride [47]. These results may suggest that tumors were responsive to decreased androgen levels via 5α-reductase inhibition, because downregulating tumor AR may slow or halt the development and progression of prostate cancer [48]. However, since these were not significant differences how much can be inferred is limited. The difference in AR expression in tumor with GU weight > 1 gram was not statistically significant between treatment and control, as previously reported [49]. This may demonstrate the absence of finasteride or dutasteride treatment-associated effect in TRAMP mice large tumor AR expression. There was a notable increase in AR levels in hyperplasia versus prostate epithelium, which is similar to the high total cytoplasmic and nuclear AR content of hyperplastic prostates versus normal prostates in 2.5- to 4.6-year-old dogs [50]. AR was mostly increased in prostate epithelium versus tumor in treatment groups, which is similar to the significant increase in AR levels in benign versus tumor in human prostate following finasteride treatment [49]. It is significant to note that benign rodent prostate tissues following androgen deprivation (castration) therapy can adapt to low serum testosterone levels to maintain functional androgen levels, which can contribute to tumorigenesis [51].

5α-reductase 1 was predominantly expressed in prostate epithelium, while 5α-reductase 2 was highly expressed in prostate epithelium and stroma, which are consistent with previous evidence in humans [12, 52]. There were no clear trends or differences in 5α-reductase 1 and 5α-reductase 2 levels between large and small tumors, which is not consistent with increased/unchanged 5α-reductase 1 and/or decreased/lost 5α-reductase 2 mRNA expression/activity in prostate cancer versus nonmalignant prostate tissues [12, 13, 53]. Even though 5α-reductase 1 has been found to increase in high grade versus low grade prostate cancer [54, 55], this study is the first to report that tumor 5α-reductase 1 levels are positively correlated with adjusted prostate lesion score severity. Previously we found that prostate epithelium 5α-reductase 1 levels were positively correlated with adjusted prostate lesion score severity [30]. Collectively these may suggest that elevated 5α-reductase 1 levels are a characteristic of the increased malignant potential of tumors with higher prostate lesion scores. This is consistent with increased 5α-reductase 1 in human prostate cancer versus normal and/or hyperplastic prostate [12, 53].

Given the small sample sizes, it is difficult to pinpoint exactly what caused the discordant response in the Pre-Dutasteride and finasteride groups with great certainty. However, upregulated 5αR1 levels in large tumors may explain the discordant response observed in the Pre-Finasteride and Pre-Dutasteride groups. It is not clear what the mechanism is for the different response in the Post-Finasteride group. Post-Dutasteride treatment was more effective than Pre-Dutasteride treatment in decreasing 5α-reductase 1 in large tumors which may explain why Post-Dutasteride treatment was more effective in decreasing PIN progression and prostate cancer development.

There are clear limitations to this study. We were unable to make some comparisons due to the lack of replicates or loss of one or more cell types in the prostate. This limited the interpretation of the results. The morphology of the negative cells, especially, was not sufficient enough to allow for the quantification of an accurate cell count. Thus, using tissue area for normalization was the best approach. Also, 5α-reductase mRNA may not fully reflect enzymatic activity. In conclusion, the effect of finasteride and dutasteride on these biomarkers did not clearly elucidate their mechanism of action, but tumor 5αR1 levels were significantly positively correlated with adjusted prostate severe lesion score.

## Declarations

### Author contribution statement

Alexander B. Opoku-Acheampong: Performed the experiments; Analyzed and interpreted the data; Contributed reagents, materials, analysis tools or data; Wrote the paper.

Jamie N. Henningson: Performed the experiments; Analyzed and interpreted the data; Contributed reagents, materials, analysis tools or data.

Brian L. Lindshield: Conceived and designed the experiments; Analyzed and interpreted the data; Contributed reagents, materials, analysis tools or data.

### Funding statement

This study was supported by National Institute of Health COBRE Epithelial Function in Health & Disease (NIH-Grant No: P20-RR017686 from NCRR) and the Johnson Center for Basic Cancer Research at Kansas State University. This study was partially supported with funds from the Kansas Agricultural Experiment Station (Contribution # 17-017-J).

### Competing interest statement

The authors declare no conflict of interest.

### Additional information

Supplementary content related to this article has been published online at http://dx.doi.org/10.1016/j.heliyon.2017.e00360.

No additional information is available for this paper.
