# Supplementary material for: The impact of finasteride and dutasteride treatments on proliferation, apoptosis, androgen receptor, 5α-reductase 1 and 5α-reductase 2 in TRAMP mouse prostates
Source: Heliyon. 2017 Jul 24;3(7):e00360. doi: 10.1016/j.heliyon.2017.e00360 (PMC5526468; doi:10.1016/j.heliyon.2017.e00360)
Supplement: Appendix [file mmc1.docx]

Appendix

Table 1. Ki-67 expression in finasteride and dutasteride treated TRAMP. The values are the mean total Ki-67 positive cells per tissue area (µm^2^) ± SEM in prostate epithelium, hyperplasia, or tumor. Data are multiplied by 1000.

| TRAMP mice with GU weight < 1 gram | | | | | | |
| --- | --- | --- | --- | --- | --- | --- |
| Group | n | Prostate epithelium | n | Hyperplasia | n | Tumor |
| Control | 4 | 0.2 ± 0.2 | 4 | 0.3 ± 0.1* | 2 | 0.2 ± 0.1 |
| Pre-Finasteride | 5 | 0.4 ± 0.2 | 5 | 1.2 ± 0.9 | 2 | 0.5 ± 0.3 |
| Post-Finasteride | 5 | 0.3 ± 0.2 | 5 | 1.1 ± 0.5 | 1 | 0.9 |
| Pre-Dutasteride | 4 | 0.6 ± 0.4 | 4 | 1.7 ± 0.8 | 2 | 0 |
| Post-Dutasteride | 4 | 1.1 ± 0.7 | 5 | 0.7 ± 0.3* | 1 | 0.3 |
| TRAMP mice with GU weight > 1 gram | | | | | | |
| Control | 3 | 3.9 ± 3.3 | 2 | 2.1 ± 0.5 | 3 | 0.8 ± 0.4 |
| Pre-Finasteride | 2 | 1.0 ± 1.0^1,2^ | 2 | 2.9 ± 0.5^2^ | 4 | 0.4 ± 0.1^1^ |
| Post-Finasteride | 1 | 0 | 1 | 2.2 | 4 | 0.4 ± 0.1 |
| Pre-Dutasteride | nt | --- | nt | --- | 2 | 0.5 ± 0.4 |
| Post-Dutasteride | 3 | 0^1^ | 3 | 2.7 ± 0.5^2^ | 4 | 0.6 ± 0.3^1^ |

Values with different superscript numbers are statistically different from one another within group (*p* < 0.05).

Values with superscript asterisk are statistically different from one another between cell type within group in genitourinary weight < 1 gram versus > 1 gram, respectively (*p* < 0.05).

nt = no cell type was identified within tissue.

**Table 2.** Apoptosis expression in finasteride and dutasteride treated TRAMP mice. The values are the mean total apoptosis positive cells per tissue area (µm^2^) ± SEM in prostate epithelium, hyperplasia, or tumor. Data are multiplied by 1000.

| TRAMP mice with GU weight < 1 gram | | | | | | |
| --- | --- | --- | --- | --- | --- | --- |
| Group | n | Prostate | n | Hyperplasia | n | Tumor |
| Control | 1 | 0.2 | 5 | 0.3 ± 0.1 | 2 | 0.3 ± 0.1 |
| Pre-Finasteride | nt | --- | 4 | 0.4 ± 0.1 | 1 | 0.6 |
| Post-Finasteride | 1 | 0.3 | 5 | 0.3 ± 0.1 | 1 | 0.1 |
| Pre-Dutasteride | 1 | 0.2 | 4 | 0.5 ± 0.2^1^ | 2 | 2.0^2^ |
| Post-Dutasteride | nt | --- | 4 | 0.6 ± 0.2 | 1 | 0.7 |
| TRAMP mice with GU weight > 1 gram | | | | | | |
| Control | nt | --- | 2 | 0.7 ± 0.3 | 4 | 0.9 ± 0.4 |
| Pre-Finasteride | nt | --- | 2 | 0.3 ± 0.1 | 4 | 1.1 ± 0.3 |
| Post-Finasteride | nt | --- | nt | --- | 4 | 0.4 ± 0.1 |
| Pre-Dutasteride | nt | --- | 1 | 3.5 | 2 | 0.9 ± 0.2 |
| Post-Dutasteride | 1 | 0.5 | 1 | 1.2 | 4 | 0.4 ± 0.1 |

Values with different numbers are statistically different from one another within group (*p* < 0.05)

nt = no cell type was identified within tissue.

Table 3. Androgen receptor expression in finasteride and dutasteride treated TRAMP mice. The values are the mean total androgen receptor positive cells per tissue area (µm^2^) ± SEM in prostate epithelium, hyperplasia, or tumor. Data are multiplied by 1000.

| TRAMP mice with GU weight < 1 gram | | | | | | |
| --- | --- | --- | --- | --- | --- | --- |
| Group | n | Prostate | n | Hyperplasia | n | Tumor |
| Control | 5 | 5.2 ± 0.5^1^ | 5 | 9.6 ± 0.4^a,2^ | 1 | 8.8 |
| Pre-Finasteride | 5 | 6.8 ± 0.5^1^ | 5 | 7.0 ± 0.7^b,1^ | 2 | 1.7 ± 1.5^2^ |
| Post-Finasteride | 5 | 7.5 ± 1.2 | 5 | 9.1 ± 0.8^a^ | 1 | 0 |
| Pre-Dutasteride | 5 | 5.7 ± 0.8^1^ | 5 | 9.1 ± 0.9^a,2^ | 2 | 2.9 ± 2.9^1^ |
| Post-Dutasteride | 5 | 5.7 ± 1.4 | 5 | 7.9 ± 0.3^a,b,^* | 1 | 5.4 |
| TRAMP mice with GU weight > 1 gram | | | | | | |
| Control | 3 | 7.7 ± 1.4^a,b,1^ | 3 | 8.8 ± 1.0^1^ | 4 | 2.1 ± 1.5^2^ |
| Pre-Finasteride | 3 | 7.3 ± 0.9^a,b,1^ | 3 | 8.9 ± 0.9^1^ | 4 | 0.4 ± 0.2^2^ |
| Post-Finasteride | 4 | 6.6 ± 0.2^a,1^ | 4 | 8.9 ± 1.0^2^ | 4 | 0.2 ± 0.1^3^ |
| Pre-Dutasteride | 1 | 9.8 | 1 | 8.9 | 2 | 1.5 ± 1.4 |
| Post-Dutasteride | 4 | 9.3 ± 0.7^b,1^ | 4 | 9.6 ± 0.6^1^ | 4 | 0.7 ± 0.5^2^ |

Values with different letters are statistically different from one another between groups (*p* < 0.05).

Values with different numbers are statistically different from one another within group (*p* < 0.05).

Values with asterisk are statistically different from one another between cell type within group in genitourinary weight < 1 gram versus > 1 gram, respectively (*p* < 0.05).

**Table 4.** 5α-reductase 1 expression in finasteride and dutasteride treated TRAMP mice. The values are the mean total 5α-reductase 1 probe copies per tissue area (µm^2^) ± SEM in prostate epithelium, hyperplasia, or tumor.

| TRAMP mice with GU weight < 1 gram | | | | | | |
| --- | --- | --- | --- | --- | --- | --- |
| Group | n | Prostate | n | Hyperplasia | n | Tumor |
| Control | 5 | 4.0 ± 1.1^a^ | 5 | 5.9 ± 1.5 | 1 | 12.4 |
| Pre-Finasteride | 5 | 8.7 ± 3.6^a,b^ | 5 | 6.9 ± 3.5 | 3 | 4.2 ± 1.3* |
| Post-Finasteride | 5 | 22.2 ± 8.5^b^ | 5 | 14.8 ± 6.0 | 2 | 19.4 ± 9.6 |
| Pre-Dutasteride | 3 | 11.1 ± 7.4^a,b^ | 3 | 6.2 ± 4.4 | 2 | 13.4 ± 7.2* |
| Post-Dutasteride | 5 | 13.7 ± 5.3^a,b^ | 5 | 5.2 ± 1.4 | nt | --- |
| TRAMP mice with GU weight > 1 gram | | | | | | |
| Control | nt | --- | 1 | 3.8 | 4 | 22.7 ± 4.7^a^ |
| Pre-Finasteride | nt | --- | nt | --- | 4 | 24.0 ± 2.2^a^ |
| Post-Finasteride | 1 | 29.1 | 1 | 15.6 | 4 | 28.5 ± 11.6^a,b^ |
| Pre-Dutasteride | 1 | 4.7 | 1 | 7.6 | 2 | 54.0 ± 1.2^b^ |
| Post-Dutasteride | 1 | 35.1 | 1 | 28.6 | 4 | 20.6 ± 7.2^a^ |

Values with different letters are statistically different from one another between groups (*p* < 0.05).

Values with asterisk are statistically different from one another between cell type within group in genitourinary weight < 1 gram versus > 1 gram, respectively (*p* < 0.05).

nt = no cell type was identified within tissue.

Table 5. 5α-reductase 2 expression in finasteride and dutasteride treated TRAMP mice. The values are the mean total 5α-reductase 2 probe copies per tissue area (µm^2^) ± SEM in prostate epithelium, hyperplasia, or tumor.

| TRAMP mice with GU weight < 1 gram | | | | | | |
| --- | --- | --- | --- | --- | --- | --- |
| Group | n | Prostate | n | Hyperplasia | n | Tumor |
| Control | 5 | 2.3 ± 1.3^a,1^ | 5 | 2.5 ± 1.0^1^ | 3 | 172.1 ± 118.6^2^ |
| Pre-Finasteride | 5 | 2.7 ± 1.3^a^ | 5 | 4.2 ± 0.9 | 2 | 3.1 ± 3.0 |
| Post-Finasteride | 5 | 9.5 ± 7.9^a,b,1^ | 5 | 8.6 ± 4.1^1^ | 3 | 146.0 ± 70.0^2^ |
| Pre-Dutasteride | 4 | 27.8 ± 15.4^b^ | 3 | 9.5 ± 6.0 | 2 | 4.5 ± 4.0 |
| Post-Dutasteride | 5 | 13.0 ± 5.7^a,b^ | 5 | 10.0 ± 1.8 | nt | --- |
| TRAMP mice with GU weight > 1 gram | | | | | | |
| Control | nt | --- | nt | --- | 4 | 26.8 ± 11.5 |
| Pre-Finasteride | nt | --- | nt | --- | 4 | 21.6 ± 6.6 |
| Post-Finasteride | nt | --- | nt | --- | 4 | 6.8 ± 3.0 |
| Pre-Dutasteride | 1 | 13.1 | 1 | 10.9 | 2 | 28.1 ± 19.6 |
| Post-Dutasteride | nt | --- | nt | --- | 4 | 6.9 ± 4.4 |

Values with different letters are statistically different from one another between groups (*p* < 0.05).

Values with different numbers are statistically different from one another within group (*p* < 0.05).

nt = no cell type was identified within tissue.
